# Reading component skills in dyslexia: word recognition, comprehension and processing speed

**DOI:** 10.3389/fpsyg.2014.01339

**Published:** 2014-11-28

**Authors:** Darlene G. de Oliveira, Patrícia B. da Silva, Natália M. Dias, Alessandra G. Seabra, Elizeu C. Macedo

**Affiliations:** ^1^Developmental Disorders Program, Center for Health and Biological Science, Mackenzie Presbyterian UniversitySao Paulo, Brazil; ^2^Educational Psychology Post-graduation Program, FIEO University CenterOsasco, Brazil

**Keywords:** phonological processing, reading disability, cognitive assessment, reading model, processing speed

## Abstract

The cognitive model of reading comprehension (RC) posits that RC is a result of the interaction between decoding and linguistic comprehension. Recently, the notion of decoding skill was expanded to include word recognition. In addition, some studies suggest that other skills could be integrated into this model, like processing speed, and have consistently indicated that this skill influences and is an important predictor of the main components of the model, such as vocabulary for comprehension and phonological awareness of word recognition. The following study evaluated the components of the RC model and predictive skills in children and adolescents with dyslexia. 40 children and adolescents (8–13 years) were divided in a Dyslexic Group (DG; 18 children, MA = 10.78, SD = 1.66) and control group (CG 22 children, MA = 10.59, SD = 1.86). All were students from the 2nd to 8th grade of elementary school and groups were equivalent in school grade, age, gender, and IQ. Oral and RC, word recognition, processing speed, picture naming, receptive vocabulary, and phonological awareness were assessed. There were no group differences regarding the accuracy in oral and RC, phonological awareness, naming, and vocabulary scores. DG performed worse than the CG in word recognition (general score and orthographic confusion items) and were slower in naming. Results corroborated the literature regarding word recognition and processing speed deficits in dyslexia. However, dyslexics can achieve normal scores on RC test. Data supports the importance of delimitation of different reading strategies embedded in the word recognition component. The role of processing speed in reading problems remain unclear.

## INTRODUCTION

Cognitive psychology has provided important insights concerning learning and educational issues. In this sense, this area can closely contribute to educational psychology, improving our understanding about the functioning of cognitive systems by providing theoretical models of important processes, such as reading comprehension (RC). In fact, cognitive psychologists have sought to determine which skills are involved in this complex process, and one of these models was suggested by Gough and Tunmer (1986), in which RC is a final product of the interaction between decoding and linguistic comprehension (LC) skills. This model, called the simple view of reading (SVR), reveals the componential nature of reading.

According to Gough and Tunmer (1986), the first component of SVR is decoding (D), which can be understood as the conversion of graphic symbols into sounds, during either reading aloud or silent reading. The second component of reading is LC, a general skill, i.e., non-specific to written language, and refers more specifically to the understanding of oral language (Kershaw and Schatschneider, 2010). This ability allows for the understanding of an auditory stimulus and the text comprehension process involved in reading. When both abilities are preserved, a written stimulus can be decoded and understood, and then RC occurs. If one of the processes is-9 impaired, RC will not occur, given the nature of the interaction proposed by Gough and Tunmer (1986; D × LC = RC).

Despite being considered simple, the SVR model offers a general understanding of the RC process and has been corroborated by several studies, even in different languages, despite some particularities associated with orthographic transparency (Aaron et al., 1999, 2008; Betjemann et al., 2008; Florit and Cain, 2011). At the same time, it is conceivable that the components of the SVR could be decomposed and involve other abilities (Kirby and Savage, 2008), and in fact, these authors call it a reductionist model. Some suggestions for changes and additions have been made, especially regarding RC strategies, the importance of the fluency and the influence of illustrations during reading in LC component.

One of these proposals refers to the decoding component. Researchers suggest that decoding could be better understood in terms of word recognition (Aaron et al., 1999, 2008; Kirby and Savage, 2008; Dias et al., 2014). Such a change could allow for some theoretical integration. For example, Frith (1985, 1997) describes three different strategies that one can use for word recognition. The first one is called the logographic strategy and is characterized by the use of contextual cues in recognition, with the reader interpreting words as drawings. Reading is an overall visual recognition act. The second strategy is the alphabetical strategy. It emerges as the reader learns correspondence rules between graphemes and phonemes and starts to use decoding/coding in reading and writing. Finally, the third strategy, the orthographic strategy, emerges due to the reader’s accumulated reading experience and the development of a mental orthographic lexicon, becoming able to read familiar words through direct recognition. The development of these strategies, their use, and the relationship with school performance have already been shown in Brazilian students throughout the course of elementary school, corroborating the model for Portuguese, which has a relatively transparent orthography (Dias et al., accepted).

Another proposal for the SVR model refers to a third component, in addition to word recognition and LC. Research has suggested that verbal processing speed integrates the component processes of RC (Aaron et al., 1999, 2008). Processing speed has been related to automaticity and fluency (Georgiou et al., 2013). In this sense, when a process becomes automatic, it demands less cognitive effort, so resources can be redirected to allow for the deepest levels of comprehension about what one is reading (Norton and Wolf, 2012). Thus, processing speed appears to add a little variation to reading performance that is not explained by word recognition and LC. The dissociation can be seen in Cardoso-Martins and Pennington’s (2001) study, which showed that processing speed, as assessed by rapid automatized naming (RAN), can contribute with unique variance to reading/writing, independently of phonological awareness. Most interestingly, phonological awareness was an important predictor of decoding skills, since this construct relates to recognition and manipulation of different levels of speech components like words, syllables, and phonemes (Santos and Navas, 2002). On the other side, RAN tasks may be better predictors of reading fluency than measures of phonemic awareness because they assess the fluency of fundamental cognitive processes required for construction of sight word representations (Torgesen and Hudson, 2006). Furthermore, RAN tests may measure other cognitive skills, like phonemic sequencing, rapid articulatory planning, lexical sequencing, etc. (Aguilar-Vafaie et al., 2012). Corroborating these findings, Norton and Wolf (2012) reviewed a series of studies and concluded that RAN and phonological awareness, despite being predictors of reading, should be considered distinct constructs. As phonological awareness is a cognitive skill assessed in cases of learning difficulties, RAN tests are also very commonly applied in this scenario (Kirby et al., 2010).

There is still a discussion about fluency as an independent factor, considering it the result of competence or automation in word recognition. Several studies have suggested that there are specific aspects of fluency that are relatively independent of the word recognition (Adlof et al., 2006; Collins and Levy, 2008; Joshi and Aaron, 2000; Zadeh et al., 2010; Seabra et al., 2012). It is noteworthy that Georgiou et al. (2013) found a high correlation between skills measured by RAN test and reading fluency, as both are important skills for reading speed. A longitudinal study with Dutch children (De Jong and van der Leij, 2003) similarly found that whereas 6th grade dyslexic and normally reading groups were very different in their reading speed or reading level there was considerable overlap on decoding accuracy. This relative dissociation between reading accuracy and reading fluency among dyslexic students learning to read languages with more transparent orthographies is interesting in light of the difficulties we have just described in “closing the gap” in reading fluency with older children with reading disabilities. In both cases, after effective instruction, students with reading disabilities are more similar to average readers in their phonemic decoding and reading accuracy scores than they are in reading fluency.

Another detail of the SVR is the consideration that the model components can be decomposed and involve other abilities. For example, a large amount of evidence indicates that an important ability for word recognition is phonological processing, mainly phonological awareness, or the ability to reflect on and manipulate speech sounds (Aaron et al., 2008; Capovilla and Dias, 2008; Cuadro and Trías, 2008; Shaywitz et al., 2008; Fletcher et al., 2009; Seabra and Dias, 2012). Evidence from experimental studies corroborates a causal relationship between phonological awareness and word recognition skills (Schatschneider and Torgesen, 2004; O’Connor et al., 2005; Cuadro and Trías, 2008; Simmons et al., 2008). On the other hand, LC includes vocabulary, syntax, morphology, semantics, ability to generate inferences and construct mental schemes, among other verbal skills, in addition to attention, working memory and reasoning (Kirby and Savage, 2008; Morais et al., 2013). Oral vocabulary is considered one of the main predictors of RC, with a strong correlation between them. It is noteworthy that the vocabulary to be understood in reading is the same used in oral language (Biemiller, 2006). In brief, there is evidence that comprehension processes and word recognition share some variance, but at the same time, there are abilities that contribute with unique variance to each one, such as the vocabulary to comprehension and phonological awareness to word recognition (Seabra and Dias, 2012).

Thus far, the cognitive model of reading could be summarized as the interaction between (1) word recognition, which can occur by logographic, alphabetic, and orthographic strategies (not only decoding), in which phonological awareness has an important role; (2) LC, in which vocabulary appears to exert important unique variance; and (3) processing speed (a new suggested component). This model does not claim to be complete because other authors have suggested, for example, a main contribution of working memory skills in RC (Carretti et al., 2009). Beyond that, there are additional psychological components that can also affect the RC process, such as motivation, intelligence, and locus of control, along with environmental factors, such as genre and cultural, linguistic, social, scholarly, and familiar contexts, as suggested in the Component Reading Theory (Aaron et al., 2008). However, in this work, our interest is limited to the cognitive components of the RC model.

Applying this model to dyslexic children can give us some insights about the specific deficits present, in terms of components or underlying abilities. Developmental dyslexia is a neurodevelopmental disorder of biological origin, generating behavioral signs that mainly affect reading accuracy and fluency [Frith, 1997; American Psychiatric Association (APA), 2003; Lyon, 2003]. Individuals with dyslexia perform poorly on tasks that require phonological awareness, verbal short-term memory, and lexical access. Performance on these phonological tasks predicts reading acquisition in both normal and dyslexic readers (Norton and Wolf, 2012). Additionally, due to their difficulties in processing visual and auditory information quickly, as well as lexical access, dyslexic subjects have difficulties in processing speed and RAN, as these are skills that reflect the time at which the information is processed in the brain with the integration of visual stimuli with linguistic functions (Wolf and Denckla, 2005). In this sense, one could hypothesize that LC in dyslexics is relatively preserved but that there are deficits in word recognition due to the underlying impairment in phonological awareness, in addition to difficulties in processing speed (Wolf and Denckla, 2005; Norton and Wolf, 2012). These deficits impact the results of RC. Indeed, Wolf and Bower (1999) developed a paradigm called the double deficit hypothesis, in which they describe naming or processing speed and phonological awareness as important skills for the development of reading. Thus, individuals with learning disabilities in reading could have deficits in one or both of these skills, as they are extremely important for reading competence. RAN and phonological awareness are both important predictors of reading ability, even in the early stages of learning, and one or both are often impaired in dyslexia (Norton and Wolf, 2012).

The aim of this study was to investigate the components of the RC model and predictive skills in a group of Brazilian children and adolescents with developmental dyslexia. In this sense, listening, RC, and word recognition skills were assessed, considering the logographic, alphabetic and orthographic strategies, processing speed, picture naming, receptive vocabulary and phonological awareness. Based on the literature, we hypothesized that the LC component and related skills (vocabulary) would be preserved. Concerning the word recognition component, it will most likely be impaired in this sample, as well as the underlying ability of phonological awareness. Lastly, we believe that the third suggested component, processing speed, will be impaired in this sample.

## MATERIALS AND METHODS

### PARTICIPANTS

Forty children and adolescents (8–13 years old), 29 boys (72.5) and 11 girls (27.5 %), all students from the 2nd to the 8th grade of elementary school, participated in the study. The dyslexic group (DG) comprised 18 children (72.2% boys) diagnosed with dyslexia by multidisciplinary teams. Diagnostics were based on DSM IV-TR [American Psychiatric Association (APA), 2003]. Twenty-two normal readers (72.7% boys) comprised the control group (CG). All participants were assessed using a neuropsychological battery, including reading, writing and phonological awareness tests. Moreover, the groups were equivalent in terms of school grade (*X^2^* = 6.227; *p* = 0.398), gender (*X^2^* = 0.001; *p* = 0.972), type of school (public x private; *X^2^* = 0.258; *p* = 0.611), age, and IQ (**Table 1**).

**Table 1:** Dyslexic and control groups’ ages and IQs, with *t* and *p* statistics.

Measure	Dyslexic group Mean (SD)	Control group Mean (SD)	*t*	*p*
**Age**	10.78 (1.66)	10.59 (1.86)	0.341	0.735
**Full IQ**	112.36 (9.57)	116.38 (14.13)	-0.898	0.377

The inclusion criteria for the DG in the present study was presence of significant deficits based on the discrepancy between expected and attained reading skills according to the DSM-IV-TR [American Psychiatric Association (APA), 2003]: 2 SD below in Words and Pseudowords Reading Competence Test (WPRCT), a Brazilian neuropsychological word reading test (Capovilla and Seabra, 2010). The inclusion criteria for the CG were the same to as the DG group for age, IQ and absence of associated medical conditions. However, the CG participants did not present low performance in reading competence tests and had no history of learning difficulties. Participants with clinical, neurological or psychiatric disorders, sensory deficits, attention deficit hyperactivity disorder (ADHD) or other developmental disorders were not included.

### MATERIALS

A semi-structured interview was conducted with all participants’ parents to exclude the presence of clinical, neurological or psychiatric disorders (including ADHD), as well as the presence of sensory deficits or other developmental disorders. Intelligence level was assessed by the Brazilian third version of the Wechsler Intelligence Scale for Children (WISC-III; Figueiredo, 2001). Oral and written language skills were assessed with the following instruments. All tests had acceptable psychometric features for Brazilian children and teenagers.

Writing Sentences Comprehension Test (WSCT; Macedo et al., 2005): computerized test that assesses meaning extracted from written sentences with different difficulty levels. The test consists of six figures representing one sentence written above them. In each item, the subject should read the written sentence and click on the figure that corresponds to the read sentence.

Oral Sentences Comprehension Test (OSCT; Macedo et al., 2005): computerized test that assesses meaning extracted from listened sentences with different difficulty levels. Each item of the test presented six pictures and one spoken sentence, and only one picture matched the sentence. The subject must click on the picture that best explains the spoken sentence.

Reading Competence Test (RCT; Macedo et al., 2005): computerized test that verifies word recognition skills and strategies via judgment of pictures and written word pairs (pressing a ‘correct’ or ‘incorrect’ button). The word categories included (1) Regular Correct words [RC, e.g., the word ‘FADA’ (fairy in English) on the figure of a fairy], (2) Irregular Correct words [IC, e.g., the word ‘TÁXI’ (taxi) on the figure of a taxi], (3) Semantic Confusion [SC, there is a semantic change, e.g.: the word ‘LARANJA’ (orange) on the picture of a banana], and pseudowords derived from real words, as (4) Orthographic confusion [OC, pseudoword with orthographic errors, but correct phoneme-grapheme conversion, e.g., the word ‘OSPITAL’ (the correct way to write it in Portuguese is HOSPITAL) on the figure of a hospital], (5) Visual Confusion [VC, e.g., the word ‘CAEBÇA’ (the correct way to write it in Portuguese is CABEÇA) on the figure of a head], (6) Phonological Confusion [PhC, e.g., the word ‘MÁCHICO’ (the correct way to write it in Portuguese is MÁGICO) on the figure of magic], and (7) Weird pseudoword [WNw, pseudoword not derivate from a real word, e.g., the word ‘RASSUNO’ (a word that does not exist in Portuguese) on the figure of a hand].

Peabody Picture Vocabulary Test (PPVT; Dunn and Dunn, 2007): a computerized test that assesses receptive vocabulary. Words are spoken, and then the subject must click on the figure representative of the meaning of the word.

Phonological Awareness Test (PhoAT; Macedo et al., 2005): a computerized test that assesses the ability to manipulate speech sounds through tasks involving rhyme, alliteration, phonemic addition, phonemic subtraction, and phonemic transposition.

Picture Naming Test (PNT; Strauss et al., 2006): the test consists of four images of objects: house, elephant, ball, watch. The subject must name each item as fast as possible in linear order from line by line. The number of errors and the total completion time were computed.

### PROCEDURES

The participants’ parents gave their written informed consent to take part in the study, which was approved by the Institutional Review board. All individuals participated voluntarily. The testing took place over three sessions, each with an average duration of 1 h and 30 min. The tests were randomly administered.

### STATISTICAL ANALYSES

Multivariate analysis of variance was performed for all measurements to determine any group differences in performance. Group (DG × CG) was used as the independent variable, and the measures (in terms of score and time) of listening and RC, word recognition, considering the logographic, alphabetic and orthographic strategies, picture naming, receptive vocabulary and phonological awareness were dependent variables. The level of confidence was set at 0.05 for all of the comparisons. Significant results are highlighted in bold. The table also reports the partial eta square.

## RESULTS

Descriptive and inferential statistics are presented in **Table 2**. The groups did not differ in measures of writing and oral sentence comprehension, phonological awareness, naming, and vocabulary. In terms of score, a significant group effect was found for the RCT, in which the DG presented worse performance. The analysis suggested that the DG individuals had the most difficulty with the OC items of the RCT. In terms of time, the MANOVA results revealed significant differences between groups in the time required to the Picture Naming Test, in which the DG required more time to complete the tasks. In brief, the DG performed worse than the CG only in word recognition, and required more time to complete the processing speed task.

**Table 2:** Descriptive and inferential statistics for children’s performances on each measurement with F and *p* values for the Dyslexic (DG) and Control (CG) Groups.

	Group	Mean	SD	F	*p*	Partial eta square
WSCT	DG	35.47	5.65	(1, 31) = 0.902	0.349	0.028
	CG	37.19	4.65			
OSCT	DG	38.29	1.72	(1, 31) = 0.686	0.414	0.022
	CG	37.69	2.44			
**RCT**	**DG**	**59.53**	**4.80**	**(1, 31) = 4.505**	**0.042**	**0.127**
	**CG**	**63.50**	**5.92**			
RC	DG	9.65	0.61	(1, 31) = 0.678	0.417	0.021
	CG	9.81	0.54			
IC	DG	8.76	1.39	(1, 31) = 1.581	0.218	0.049
	CG	9.25	0.68			
SC	DG	9.82	0.53	(1, 31) = 0.387	0.533	0.013
	CG	9.69	0.70			
**OC**	**DG**	**5.65**	**2.21**	**(1, 31) = 4.551**	**0.041**	**0.128**
	**CG**	**7.63**	**3.07**			
VC	DG	9.12	0.93	(1, 31) = 1.716	0.200	0.052
	CG	9.50	0.73			
PhC	DG	6.53	2.50	(1, 31) = 1.433	0.240	0.044
	CG	7.63	2.75			
WNw	DG	10.00	0.500	(1, 31) = 0.000	1.00	0.000
	CG	10.00	0.000			
PPVT	DG	96.47	8.80	(1, 31) = 0.376	0.554	0.012
	CG	94.22	11.66			
PhoAT	DG	42.29	11.411	(1, 31) = 2.458	0.127	0.073
	CG	49.25	14.017			
PNT	DG	1.07	1.58	(1.33) = 0.552	0.463	0.017
	CG	0.72	1.07			
**PNT_time**	**DG**	**46.07**	**14.58**	**(1.33) = 9.216**	**0.005**	**0.229**
	**CG**	**33.78**	**8.34**			

*PNT time – seconds.*

## DISCUSSION

The results showed that dyslexic children and teenagers have deficits related to word recognition, as expected [Frith, 1997; American Psychiatric Association (APA), 2003; Lyon, 2003]. Specifically, the data revealed a specific deficit in orthographic strategy, as indicated by the pattern of errors in the OC items of the RCT. This finding suggests that dyslexics have more difficulty in composing an orthographic mental lexicon, and consequent trouble with the use of the orthographic strategy of reading. This result could be due to the presence of older children and teenagers in our sample; that is, these participants could have learned decoding skills, despite the persistence of difficulties in orthographic lexicon. Our sample is limited in number to provide more detailed analyses about reading strategies in more homogenous age clusters. Our dyslexics showed appropriate phonological awareness, vocabulary, naming, oral, and RC accuracy. Despite this, the dyslexics required more time to perform the naming test, which can be related to processing speed (Wolf and Bower, 1999; Wolf and Denckla, 2005).

Concerning the modified SVR (WR × LC = RC, as the Gough and Tunmer, 1986; revised formula, Frith, 1985, 1997; Aaron et al., 1999, 2008; Kirby and Savage, 2008; Dias et al., 2014), that replaces the component ‘decoding’ for ‘word recognition,’ dyslexics presented poor word recognition skills with specific deficits in orthographic reading. This data could suggest that the formula should be further specified to consider all the reading strategies. Models should try to decompose the WR component and investigate the interactions between reading strategies that result in the WR index. Possibly different strategies could have different weights to the word recognition component and could impact RC also in different ways. For example, individuals with difficulty in using the alphabetic strategy usually have a delay in the development of the orthographic strategy, and such pattern of difficulty in two strategies could influence the word recognition component, leading to consistent problems with the result of the formula, RC. On the other hand, with only the orthographic strategy impaired, word recognition could happen with the alphabetic strategy, keeping a reasonable accuracy in RC, but expanding more time due to the underlying decoding processes (Frith, 1985, 1997).

Furthermore, besides preserved logographic and alphabetic reading, dyslexics performed the same as controls in the oral comprehension test, suggesting they have adequate oral language skills to understand the context and make inferences, including preserved vocabulary skills. Therefore, it makes sense that their RC skill was relatively preserved. On the other hand, processing speed was compromised in our dyslexics (Wolf and Denckla, 2005; Norton and Wolf, 2012). In this sense, if one considers processing speed as a new component of the reading model (Aaron et al., 1999, 2008), it would be expected to impact on the performance of participants on RC, which was not observed. Some hypotheses can be offered to explain this finding. For example, it is possible that the processing speed correlates in a more complex (and not direct) way with comprehension processes. It is also possible that, despite failures in processing speed, the participants have developed strategies that enable them to compensate for these difficulties, so that such slowness do not seem to impact its performance. It is possible that this lower speed has impacts on other measures, as tasks that request the individual to read with pressure of time.

Also, one must consider some specificity of the tests used. In this context, processing speed could be more important as the working memory demands of the task increase. That is, to process a greater amount of information, we need to process such information faster or we can overload our working memory capacity. In this sense, we should consider that our reading comprehension assessment WSCT uses short sentences, and sentence reading does not demand as much working memory skills as reading passages or paragraphs does. That condition might have facilitated dyslexic performance, and we think that in longer texts, the overload of working memory can impair their RC measures, so that processing speed could be a predictor for the comprehension of this type of complex reading. This hypothesis should be addressed in future research.

### FINAL VIEWS

This study suggested some specification in the reading component model and revealed specific deficits in participants with dyslexia. It was observed that they had preserved auditory comprehension but showed deficits in the word recognition component. Word recognition was also dissociated, with deficits in orthographical strategy but preservation of logographic and alphabetical reading. Such dissociation might support the components of reading model to be further specified and future research should suggest models. We also found deficits in processing speed, but these deficits did not seem to impact RC, suggesting a more complex (and not direct) influence of speed on comprehension process. We suggest that new studies use more complex RC tasks and investigate how working memory can interact with processing speed and RC. Beyond the absence of a working memory measurements and the use of a relatively simple RC task, some limitations of this study included the small number of participants and the heterogeneity of the sample, with a large range of ages and school grades.

## Conflict of Interest Statement

The authors declare that the research was conducted in the absence of any commercial or financial relationships that could be construed as a potential conflict of interest.
